# Supplementary material for: Genomic Selection for Any Dairy Breeding Program via Optimized Investment in Phenotyping and Genotyping
Source: Front Genet. 2021 Feb 10;12:637017. doi: 10.3389/fgene.2021.637017 (PMC7928407; doi:10.3389/fgene.2021.637017)
Supplement: Supplementary file 1 [file Data_Sheet_1.docx]

Supplementary Material

# Supplementary Figures and Tables

## Supplementary Tables

**Table S1 Accuracy of conventional and genomic selection with varying number of phenotypes and phenotyped animals.** NoRec = Number of phenotypic records per lactation, NoDaughters = number or daughters per sire, r_sire_ = accuracy for sires, r_cows_ = accuracy for cows, r_non-pheno_ = accuracy for non-phenotyped animals, NoPhenoCows = number of phenotyped cows, NoPhenoTotal = total number of phenotypes (number of phenotypes per lactation times the number of phenotyped cows).

| NoPheno | NoDaughters | r_sires_ | r_cows_ | r_non-pheno_ | NoPhenoCows | NoPhenoTotal |
| --- | --- | --- | --- | --- | --- | --- |
| **Conventional selection, 100 sires** | | | | | | |
| Variable resources for phenotyping | | | | | | |
| 1 | 100 | 0.93 | 0.62 | 0.56 | 10,000 | 10,000 |
| 2 | 100 | 0.96 | 0.70 | 0.59 | 10,000 | 20,000 |
| 5 | 100 | 0.97 | 0.81 | 0.64 | 10,000 | 50,000 |
| 10 | 100 | 0.98 | 0.89 | 0.66 | 10,000 | 100,000 |
| Fixed resources for phenotyping | | | | | | |
| 1 | 1000 | 0.99 | 0.63 | 0.59 | 100,000 | 100,000 |
| 2 | 500 | 0.99 | 0.71 | 0.61 | 50,000 | 100,000 |
| 5 | 200 | 0.99 | 0.82 | 0.64 | 20,000 | 100,000 |
| 10 | 100 | 0.98 | 0.89 | 0.66 | 10,000 | 100,000 |
| **Genomic selection** | | | | | | |
| Variable resources for phenotyping | | | | | | |
| 1 | - | - | 0.62 | 0.56 | 10,000 | 10,000 |
| 2 | - | - | 0.70 | 0.63 | 10,000 | 20,000 |
| 5 | - | - | 0.81 | 0.71 | 10,000 | 50,000 |
| 10 | - | - | 0.89 | 0.76 | 10,000 | 100,000 |
| Fixed resources for phenotyping | | | | | | |
| 1 | - | - | 0.63 | 0.93 | 100,000 | 100,000 |
| 2 | - | - | 0.71 | 0.90 | 50,000 | 100,000 |
| 5 | - | - | 0.82 | 0.84 | 20,000 | 100,000 |
| 10 | - | - | 0.89 | 0.76 | 10,000 | 100,000 |

**Table S2 Genetic gain by scenario, relative cost of phenotyping to genotyping ($P:$G), and availability of an initial training population.** The table presents the means and standard deviations (subscript) across 10 replicates for the conventional (C) and genomic (G) scenarios, with numbers indicating the number of phenotypic records per lactation. For the $P:$G we compared the cost of 11 phenotypic records per lactation to the cost of one genotype. The scenarios in bold did not spend all the available resources. Lower-case letters denote statistically significant differences between scenarios within the same $P:$G and upper-case letters between different $P:$G within the same scenario.

|  |  | Relative cost of phenotyping (P) to genotyping (G) | | |
| --- | --- | --- | --- | --- |
|  | Scenario | $P:$G = 1:2 | $P:$G = 1:1 | $P:$G = 2:1 |
|  | C11 | 3.01_0.22_^a,A^ | 3.01_0.22_^a,A^ | 3.01_0.22_^a,A^ |
| With an initial  training population | G10 | 5.43_0.20_^b, A^ | 5.41_0.29_^b, A^ | 6.50_0.20_^b, B^ |
|  | G9 | 5.58_0.26_^b, A^ | 6.30_0.17_^c, B^ | 7.02_0.24_^c, C^ |
|  | G8 | 6.35_0.25_^c, A^ | 6.62_0.25_^d, B^ | 7.02_0.17_^c, C^ |
|  | G5 | 6.78_0.21_^d, A^ | 7.07_0.20_^e, B^ | **7.26_0.19_^c, B^** |
|  | G2 | 7.13_0.29_^e, A^ | **7.33_0.26_^e, A^** | **7.28_0.17_^c, A^** |
|  | G1 | **7.11_0.16_^e,A^** | **7.27_0.28_^e, A^** | **7.24_0.22_^c,A^** |
| Without an initial training population | G10 | 3.93_0.22_^b, A^ | 4.54_0.14_^b, B^ | 5.61_0.25_^b, C^ |
|  | G9 | 4.64_0.18_^c, A^ | 5.75_0.28_^c, B^ | 6.52_0.17_^c, C^ |
|  | G8 | 5.61_0.28_^d, A^ | 6.24_0.19_^d B^ | 6.70_0.25_^cd, C^ |
|  | G5 | 6.43_0.21_^e, A^ | 6.90_0.22_^e, B^ | **7.05_0.27_^de, B^** |
|  | G2 | 6.81_0.28_^f, A^ | **6.96_0.17_^e, A^** | **7.00_0.30_^de, A^** |
|  | G1 | **6.78_0.29_^f,A^** | **6.92_0.26_^e, A^** | **7.01_0.23_^e,A^** |

**Table S3 Intensity of sire selection by scenario and relative cost of phenotyping to genotyping ($P:$G).** PA = parent average, gEBV = genomic breeding value. We are presenting the bivariate intensities for a two-stage selection. The pre‑selection step selects the animals with the highest parent average out of all available new born males to send into testing (progeny of genomical). The selection step selects the sires with the highest breeding values out of all tested males to use in artificial insemination. The scenarios are named C/G for conventional/genomic with numbers indicating the number of phenotypic records per lactation. For the $P:$G we compared the cost of 11 phenotypic records per lactation to the cost of one genotype.

|  |  | Relative cost of phenotyping (P) to genotyping (G) | | |
| --- | --- | --- | --- | --- |
|  | Scenario | $P:$G = 1:2 | $P:$G = 1:1 | $P:$G = 2:1 |
|  | C11 | 0.10 | 0.10 | 0.10 |
| With an initial  training population | G10 | 0.17 | 0.27 | 0.37 |
|  | G9 | 0.28 | 0.39 | 0.55 |
|  | G8 | 0.38 | 0.49 | 0.68 |
|  | G5 | 0.59 | 0.77 | 0.91 |
|  | G2 | 0.77 | 0.91 | 0.98 |
|  | G1 | 0.83 | 0.92 | 0.99 |
| Without an initial training population | G10 | 0.17 | 0.25 | 0.36 |
|  | G9 | 0.28 | 0.43 | 0.56 |
|  | G8 | 0.37 | 0.55 | 0.71 |
|  | G5 | 0.58 | 0.77 | 0.91 |
|  | G2 | 0.76 | 0.89 | 0.98 |
|  | G1 | 0.83 | 0.94 | 0.99 |

**Table S4 Selection accuracy by scenario, relative cost of phenotyping to genotyping ($P:$G), and the availability of an initial training population.** The table presents the means and standard deviations (subscript) across 10 replicates for the conventional (C) and genomic (G) scenarios, with numbers indicating the number of phenotypic records per lactation. For the $P:$G we compared the cost of 11 phenotypic records per lactation to the cost of one genotype. Conventional selection implemented two-stage selection for males, hence we present the accuracy of pre-selection for progeny testing (S1) and the accuracy sire selection (S2). In genomic scenarios the candidatemales were genotyped and non‑phenotyped. We also present the accuracy for sires currently used in artificial insemination (proven males), for non‑genotyped and non‑phenotyped females (candidate females), and for all active phenotyped cows and bull dams (proven females). Lower-case letters denote statistically significant differences between scenarios within the same $P:$G and upper-case letters between different $P:$G within the same scenario. Stars denote statistically significant difference between corresponding scenarios with and without an initial training population.

|  | With an initial training population | | | Without an initial training population | | |
| --- | --- | --- | --- | --- | --- | --- |
| Scenario | $P:$G=1:2 | $P:$G=1:1 | $P:$G=2:1 | $P:$G=1:2 | $P:$G=1:1 | $P:$G=2:1 |
| Candidate males | | | | | | |
| C11, S1 | 0.37_0.04_^a ,A^ | 0.37_0.04_^a,A^ | 0.37_0.04_^a,A^ | 0.37_0.04_^a,A^ | 0.37_0.04_^a,A^ | 0.37_0.04_^a,A^ |
| C11, S2 | 0.94_0.01_^b,A^ | 0.94_0.01_^b,A^ | 0.94_0.01_^b,A^ | 0.94_0.01_^b,A^ | 0.94_0.01_^b,A^ | 0.94_0.01_^b,A^ |
| G10 | 0.89_0.03_^c,A^ | 0.90_0.02_^bc,AB^ | 0.91_0.01_^bc,B^ | 0.81_0.03_^b,A *^ | 0.84_0.01_^b,B *^ | 0.87_0.01_^b,C *^ |
| G9 | 0.90_0.03_^bc,A^ | 0.91_0.02_^bc,A^ | 0.91_0.01_^bc,A^ | 0.85_0.02_^c,A *^ | 0.87_0.01_^bc,B *^ | 0.90_0.01_^bc,C *^ |
| G8 | 0.91_0.01_^bc,A^ | 0.91_0.01_^bc,A^ | 0.91_0.01_^bc,A^ | 0.86_0.01_^cd,A *^ | 0.89_0.01_^c,B *^ | 0.90_0.01_^bc,B^ |
| G5 | 0.91_0.01_^bc,A^ | 0.91_0.00_^bc,A^ | 0.91_0.01_^bc,A^ | 0.90_0.01_^d,A^ | 0.91_0.01_^c,A^ | 0.91_0.01_^c,A^ |
| G2 | 0.91_0.01_^bc,A^ | 0.91_0.00_^bc,A^ | 0.90_0.01_^bc,A^ | 0.90_0.01_^d,A^ | 0.90_0.01_^c,A^ | 0.90_0.01_^bc,A^ |
| G1 | 0.89_0.01_^c,A^ | 0.90_0.01_^c,A^ | 0.89_0.01_^c,A^ | 0.89_0.01_^cd,A^ | 0.89_0.01_^c,A^ | 0.89_0.01_^bc,A^ |
| Proven males | | | | | | |
| C11 | 0.86_0.05_^a,A^ | 0.86_0.05_^a,A^ | 0.86_0.05_^a,A^ | 0.86_0.05_^a,A^ | 0.86_0.05_^a,A^ | 0.86_0.05_^a,A^ |
| G10 | 0.75_0.04_^b,A^ | 0.75_0.03_^b,A^ | 0.73_0.05_^b,A^ | 0.67_0.08_^bc,A *^ | 0.68_0.05_^cde,A *^ | 0.67_0.06_^b,A *^ |
| G9 | 0.76_0.04_^b,A^ | 0.72_0.06_^bc,AB^ | 0.69_0.05_^c,A^ | 0.70_0.05_^b,A *^ | 0.72_0.05_^bc,A^ | 0.71_0.05_^b,A^ |
| G8 | 0.76_0.03_^b,A^ | 0.69_0.05_^cd,B^ | 0.68_0.06_^c,B^ | 0.71_0.05_^b,A *^ | 0.74_0.05_^b,A *^ | 0.70_0.07_^b,A^ |
| G5 | 0.68_0.07_^c,A^ | 0.67_0.08_^de,A^ | 0.69_0.04_^c,A^ | 0.68_0.05_^bc,A^ | 0.69_0.05_^cd,A^ | 0.69_0.03_^b,A^ |
| G2 | 0.67_0.05_^c,A^ | 0.67_0.05_^de,A^ | 0.67_0.04_^c,A^ | 0.65_0.06_^c,A^ | 0.64_0.07_^e,A^ | 0.69_0.05_^b,A^ |
| G1 | 0.66_0.06_^c,A^ | 0.63_0.05_^e,A^ | 0.67_0.04_^c,A^ | 0.67_0.04_^bc,A^ | 0.67_0.03_^de,A^ | 0.69_0.05_^b,A^ |
| Candidate females | | | | | | |
| C11 | 0.45_0.02_^a,A^ | 0.45_0.02_^a,A^ | 0.45_0.02_^a,A^ | 0.45_0.02_^a,A^ | 0.45_0.02_^a,A^ | 0.45_0.02_^a,A^ |
| G10 | 0.48_0.01_^ab,A^ | 0.48_0.01_^ab,A^ | 0.51_0.01_^b,B^ | 0.46_0.02_^ab,A *^ | 0.47_0.02_^ab,AB^ | 0.49_0.01_^b,B *^ |
| G9 | 0.49_0.02_^b,A^ | 0.50_0.01_^b,B^ | 0.52_0.01_^b,C^ | 0.47_0.02_^ab,A *^ | 0.49_0.02_^bc,B^ | 0.52_0.01_^bc,C^ |
| G8 | 0.51_0.01_^b,A^ | 0.51_0.01_^b,A^ | 0.54_0.01_^bc,B^ | 0.49_0.02_^bc,A *^ | 0.52_0.01_^cd,B^ | 0.53_0.01_^cd,C^ |
| G5 | 0.51_0.01_^bc,A^ | 0.55_0.01_^c,B^ | 0.57_0.01_^c,C^ | 0.52_0.01_^cd,A^ | 0.55_0.01_^de,B^ | 0.57_0.01_^d,C^ |
| G2 | 0.55_0.01_^cd,A^ | 0.57_0.01_^c,B^ | 0.57_0.01_^c,B^ | 0.55_0.01_^d,A^ | 0.56_0.02_^e,AB^ | 0.57_0.01_^d,B^ |
| G1 | 0.56_0.01_^d,A^ | 0.56_0.01_^c,A^ | 0.56_0.01_^c,A^ | 0.55_0.01_^d,A^ | 0.56_0.01_^e,A^ | 0.56_0.01_^d,A^ |
| Proven females | | | | | | |
| C11 | 0.48_0.03_^a,A^ | 0.48_0.03_^a,A^ | 0.48_0.03_^a,A^ | 0.48_0.03_^a,A^ | 0.48_0.03_^a,A^ | 0.48_0.03_^a,A^ |
| G10 | 0.56_0.02_^b,A^ | 0.59_0.02_^b,B^ | 0.63_0.01_^b,C^ | 0.53_0.01_^b,A *^ | 0.56_0.01_^b,B *^ | 0.61_0.01_^b,C *^ |
| G9 | 0.59_0.03_^bc,A^ | 0.63_0.02_^c,B^ | 0.70_0.01_^c,C^ | 0.57_0.02_^bc,A *^ | 0.62_0.02_^c,B^ | 0.68_0.02_^c,C *^ |
| G8 | 0.62_0.02_^c,A^ | 0.67_0.02_^c,B^ | 0.74_0.02_^d,C^ | 0.60_0.02_^c,A *^ | 0.66_0.01_^d,B^ | 0.73_0.02_^d,C^ |
| G5 | 0.70_0.02_^d,A^ | 0.77_0.01_^d,B^ | 0.79_0.02_^e,C^ | 0.69_0.02_^d,A^ | 0.76_0.01_^e,B^ | 0.78_0.02_^e,B^ |
| G2 | 0.76_0.02_^e,A^ | 0.79_0.02_^d,B^ | 0.78_0.01_^e,AB^ | 0.76_0.01_^e,A^ | 0.77_0.02_^e,A *^ | 0.77_0.01_^de,A^ |
| G1 | 0.77_0.02_^e,A^ | 0.77_0.02_^d,A^ | 0.77_0.01_^de,A^ | 0.76_0.01_^e,A^ | 0.76_0.02_^e,A^ | 0.76_0.02_^de,A^ |

## Supplementary Tables

##
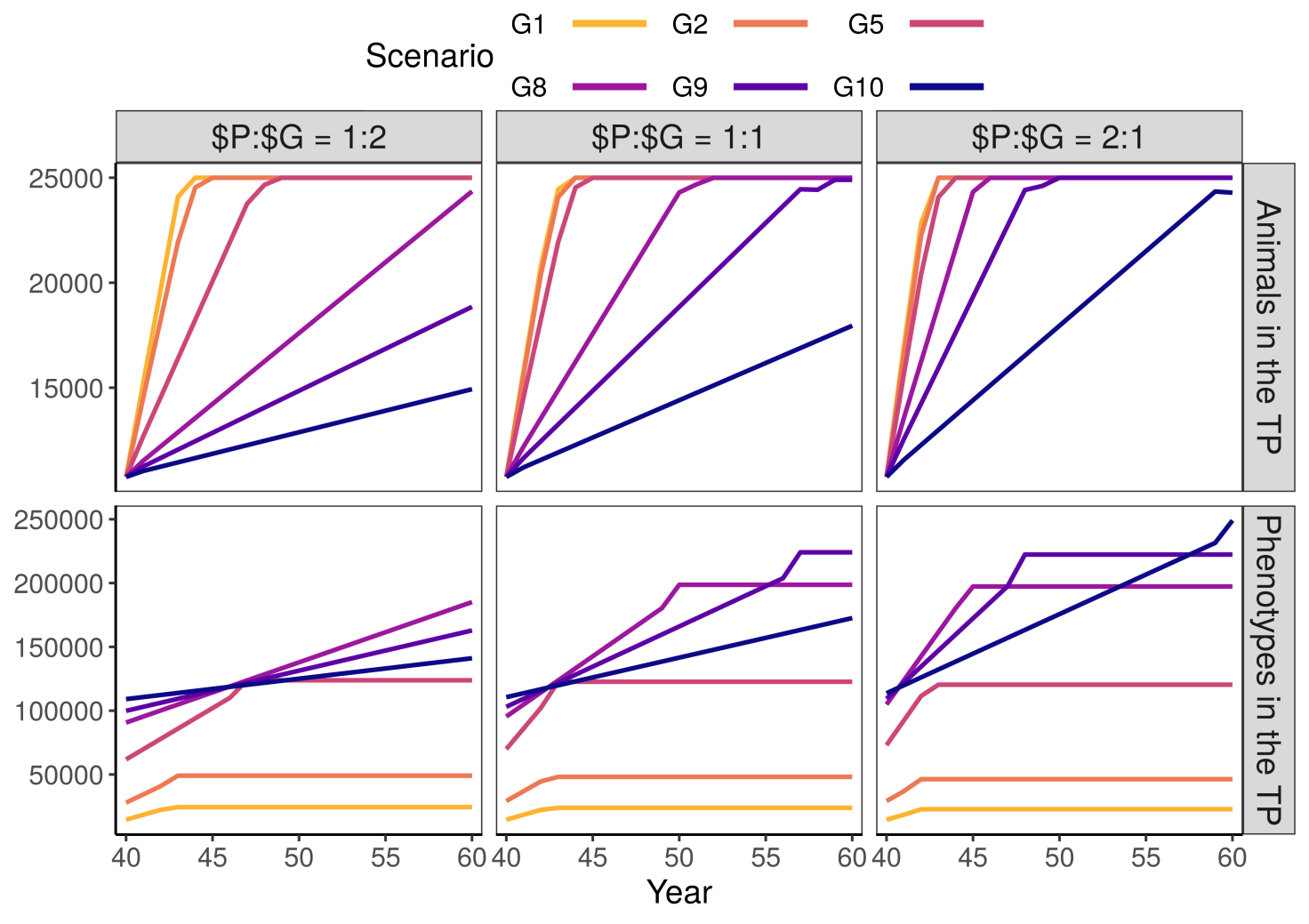


**Figure S1 The number of animals and repeated phenotypes in the training population by scenario and relative cost of phenotyping to genotyping ($P:$G) with an initial training population (TP).** The scenarios are named C/G for conventional/genomic with numbers indicating the number of phenotypic records per lactation. For the $P:$G we compared the cost of 11 phenotypic records per lactation to the cost of one genotype. In our simulation, scenarios traded repeated phenotypic records for genotypes. Hence, the scenarios with the largest training population collected the least repeated records. These were also the scenarios that achieved the highest genetic gain.
